# AprilTag array-aided extrinsic calibration of camera–laser multi-sensor system

**DOI:** 10.1186/s40638-016-0044-0

**Published:** 2016-07-25

**Authors:** Dengqing Tang, Tianjiang Hu, Lincheng Shen, Zhaowei Ma, Congyu Pan

**Affiliations:** College of Mechatronics and Automation, National University of Defense Technology, Changsha, Hunan China

**Keywords:** Multi-sensor, AprilTag array, Extrinsic calibration, Non-overlapping

## Abstract

This paper presents a new algorithm for extrinsically calibrating a multi-sensor system including multiple cameras and a 2D laser scanner. On the basis of the camera pose estimation using AprilTag, we design an AprilTag array as the calibration target and employ a nonlinear optimization to calculate the single-camera extrinsic parameters when multiple tags are in the field of view of the camera. The extrinsic parameters of camera–camera and laser–camera are then calibrated, respectively. A global optimization is finally used to refine all the extrinsic parameters by minimizing a re-projection error. This algorithm is adapted to the extrinsic calibration of multiple cameras even if there is non-overlapping field of view. For algorithm validation, we have built a micro-aerial vehicle platform with multi-sensor system to collect real data, and the experiment results confirmed that the proposed algorithm yields great performance.

## Background

Nowadays, multiple sensors are widely used in various robot systems such as unmanned ground and aerial vehicles. These sensors provide abundant information like visual image and range measurement of the environment. Fusing these sensors information is necessary to understand the environment significantly. But, whenever multiple sensors are combined, one also has to deal with additional calibration issues, which is frequently overlooked. Quantities are seldom measured at the same position and in the same coordinate frame, implying that the alignment, the relative position and/or orientation of the sensors have to be determined. A good calibration is a prerequisite to do sensor fusion.

 In many challenging tasks for robot systems like environment 3D mapping [1] and self-localization [2], cameras and 2D laser scanner supply intensity information and depth information, respectively. At the same time, a large field of view is usually required in these tasks. Capturing a large field of view is often no possible by using a single camera only, and multiple cameras have to be used. Hence, multiple cameras and 2D laser scanner will play a more and more important roles in robot systems.

**Fig. 1:**
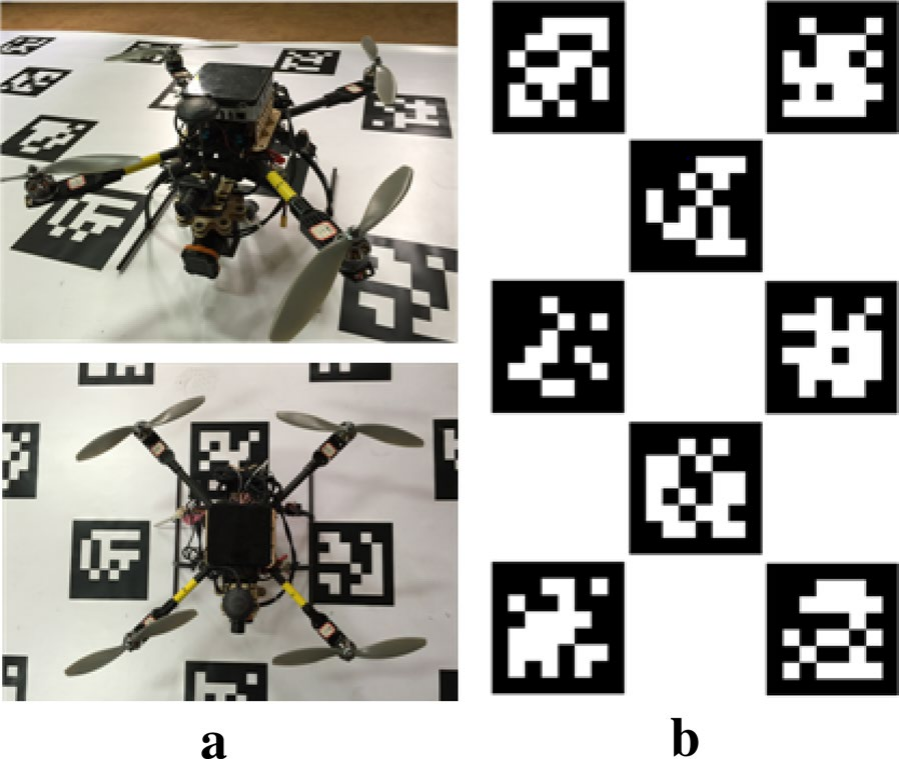
Multi-sensor system of the rotary-wing UAV and the extrinsic calibration target. **a** The rotary-wing UAV with multi-sensor system. **b** AprilTag array used as the calibration target
- We propose an extrinsic calibration algorithm between a camera and a 2D laser scanner using AprilTag array calibration target, and it is integrated with the multi-camera extrinsic calibration into a multi-sensor joint extrinsic calibration with a final global optimization.

In this paper, we suggest a convenient calibration method for a multi-sensor system including multiple cameras and a 2D laser scanner without assuming overlapping fields of view. The essential contributions of this work are the following:

- We propose an extrinsic calibration algorithm for multiple cameras, even if there is no overlapping field of view among them.
- We combine a block of tags of the AprilTag [3] into an AprilTag array to be the calibration target (see Fig. 1b) and optimize the estimated camera poses when multiple complete tags are in the field of view of a single camera.

This paper is organized as follows: Firstly, “Related work” section provides a review of related approaches. A description of extrinsic calibration pattern of multi-sensor system is given in “Calibration pattern” section. In “Extrinsic calibration of multi-sensor system” section, the joint extrinsic calibration algorithm for multi-sensor system is introduced. Experiments and discussions are presented in “Experiments” section. “Conclusion and future work” section provides the conclusion and future work.

## Related work

With the preference of multi-camera system over single camera, the extrinsic parameters calibration among multiple cameras becomes the basic requirement. In order to improve the accuracy and ignore the time-consuming of calibration, offline estimation of extrinsic parameters is desirable. In contrast to online calibration methods like [4], offline calibration relies on calibration patterns with known geometry and appearance and need not consider the real-time capability of the calibration algorithm. Conventional offline calibration uses artificial and two-dimensional calibration targets like checkerboard. It is popular because its corners can be detected accurately and reliably, while other patterns are also possibly demonstrated in [5, 6]. Svoboda et al. [7] make use of the overlapping fields of view of the cameras, and it can calibrate stereo camera or circular camera rig with all cameras pointing inwards. However, systems with cameras pointing outwards are increasingly popular, and usually there are minimal or no overlapping fields of view. Li et al. [5] presented a multi-camera system calibration toolbox adapted to minimal overlapping fields of view using a feature descriptor. In [8, 9], hand-eye calibration methods are used to calibrate this system, but they are often not accurate due to visual odometry drift. In addition to camera models, the production convenience and expansibility of the calibration pattern are also focused on. In early research, Bouguet [10] made use of cubes with a chessboard or circular dots on their surfaces. Yet this pattern is not convenient to build with high precision. Strauβ et al. [11] use checkerboard targets and combine many of them to a rigid, three-dimensional target. The checkerboards are provided with a graphical 2D code for uniquely identifying every single corner of the checkerboard. But the calibration target is also with complex structure and not easy to produce.

 Our multi-camera calibration work focuses on both the adaptability to camera models and the production convenience of calibration pattern. We group many tags of AprilTag [3] to an array and print them into a board with great planarity. These tags are identified by their different appearances, and each of them is marked as a unique ID. The extrinsic parameters of multiple cameras can be estimated only if there is one complete tag at least in each camera’s field of view.

To extrinsically calibrate a laser range finder and a camera, different calibration targets and geometry constraints are presented. Kwak et al. [12] and Li et al. [13] propose v-shaped calibration target and the right-angled triangulation board, respectively, to generate constraints on the extrinsic parameters through establishing line-point correspondences. Their drawback is that it is difficult to guarantee that the selected laser points exactly lie on the calibration target boundary. Zhang et al. [14] use the checkerboard to be the calibration target. As the state of the art, this method establishes constraints on the extrinsic parameters with plane parameters and is extended to extrinsically calibrate other kinds of range sensors and cameras [15–17]. In our research, the checkerboard was replaced with planar AprilTag array as the calibration target, and the plane-line correspondences [14] were employed to build the constraints.

## Calibration pattern

### AprilTag-based pose estimation

AprilTag is a robust and flexible visual fiducial system proposed by Olson [3] in 2011. It uses a 2D bar code style “tag” as Fig. 1a shows, allowing full 6-DOF localization of features from a single image.

**Fig. 2:**
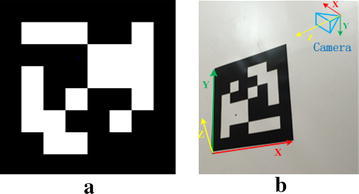
Tag used in AprilTag and the coordinate system. **a** Tag using a 2D bar cood style. **b** Tag coordinate system and camera coordinate system

This system is composed of two major components: the tag detector and the coding system. The job of the tag detector is to estimate the position of possible tags in an image using a graph-based image segmentation algorithm and then estimate the camera pose relative to the tag, that is to say computing the Euclidean transformation. The transformation between camera coordinate system and tag coordinate system is given by the transformation matrix *T*:

1 $$T = \left[ {\begin{array}{*{20}l} {R_{3 \times 3} } \hfill & \quad {t_{3 \times 1} } \hfill \\ {0_{1 \times 3} } \hfill & \quad1 \hfill \\ \end{array} } \right]_{4 \times 4}$$

where the *R*_3×3_ is a rotation matrix with *R*^*T*^*R* = *RR*^*T*^ = *I* and *t*_3×1_ is a translation vector. The Euler angles are represented by the vector *r*_3×1_=[*r*_*x*_, *r*_*y*_, *r*_*z*_]^*T*^. The tag coordinate system is presented in Fig. 2b. The coding system is to determine whether the tags are valid or not. It uses a lexicode system that can generate codes for any arbitrary tag size. There are several useful code families computed and distributed by Olson’s [3] software.

### Design of calibration target

We use part of tags of the code family 36h11 distributed by Olson [3] to generate an AprilTag array in a plane. The AprilTag array can be easily printed out in a paper with great planarity as our calibration target. The distributions of the tags in the AprilTag array are manually designed. Therefore, the transformations among all the tags are known accurately. According to Fig. 1b, we can clearly see that there is no rotation and translation along *z*-axis. The transformation matrix between arbitrary two tags (ID = *i* and ID = *j*) can be simplified as:

$$T_{i}^{j} = \left( {T_{j}^{i} } \right)^{ - 1} = \left[ {\begin{array}{*{20}l} {I_{3 \times 3} } \hfill & \quad{\begin{array}{*{20}c} {t_{x} } \\ {t_{y} } \\ 0 \\ \end{array} } \hfill \\ {0_{1 \times 3} } \hfill & \quad1 \hfill \\ \end{array} } \right]_{4 \times 4}$$

where [*t*_*x*_, *t*_*y*_, 0]^*T*^ = *t*_*i*_^*j*^.

The coordinate system of the first tag (ID = 0) in the AprilTag array is treated as the global coordinate system. Thus, the transformation matrix *T*_*n*_^0^ between an arbitrary tag (ID = *n*) coordinate system and the global coordinate system can be computed precisely without effort.

As the multi-sensor system calibration target, the AprilTag array has several advantages compared with other conventional calibration targets like checkerboards:

1. *Accurate localization* In [3], the localization accuracy of the AprilTag has been evaluated using a ray-tracing simulator. Two experiments measuring the orientation accuracy and distance accuracy validated the high precision and reliability in localization. Without loss of generality, real-world performance of the system will be lower than synthetic experiments for noise, lighting variation, etc. But it is still good.
2. *Great adaptability to camera models* The camera pose in tag coordinate system can be estimated as long as there is one complete tag in the field of view. For the multi-camera system with non-overlapping views, we can decouple these cameras into several different pairs of neighboring cameras to guarantee that at least one tag appears in fields of view. Hence, the extrinsic calibration of multiple cameras can be realized through coordinate systems transformations, which we will talk about in “Extrinsic calibration of multi-sensor system” section.
3. *Reliable tags identification* The estimated camera pose is in the tag coordinate system. Therefore, identifying these different tags in the AprilTag array is important to localize the camera in global coordinate system. The AprilTag provides the users a large number of distinguishable codes for tags and reliable tag identification algorithm.
4. *Convenient production and expansibility* The AprilTag array can be generated easily with expected size and distribution by a CDE package available at https://github.com/ethz-asl/kalibr/wiki/downloads.

Furthermore, we can conveniently extend the AprilTag array by printing additional part and then jointing the original target.

## Extrinsic calibration of multi-sensor system

The extrinsic calibration of multiple sensors is to identify the rigid transformations among these sensor coordinate systems. This paper focuses on the extrinsic calibration of a multi-sensor system including multiple cameras and a 2D laser scanner. This calibration process can be carried out in both static and dynamic environments. The static environment is advised since the dynamic environment may lead to lower accuracy due to the multiple-sensor data unsynchronization problem. In general, the calibration is pre-operated before the final task and a static environment can be easily established. Therefore, most calibration is operated under a static environment.

This calibration is composed of two components: camera to camera and camera to 2D laser scanner. These two calibration processes are combined as a joint procedure.

### Multiple-camera extrinsic calibration

**Fig. 3:**
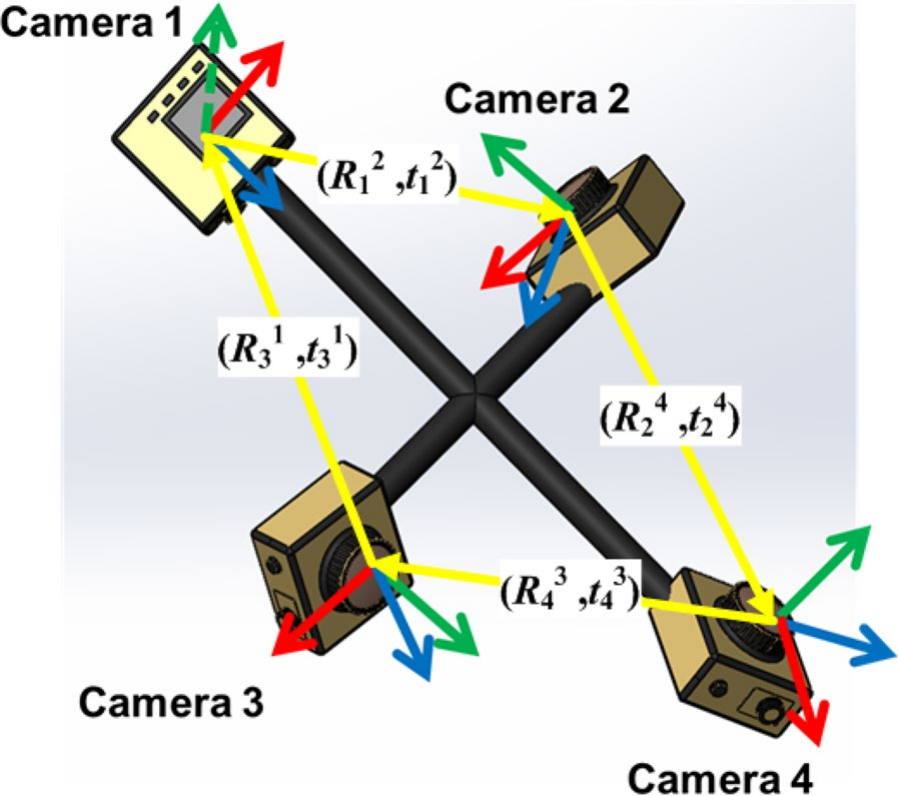
Neighboring camera pairs of multi-camera system

**Fig. 4:**
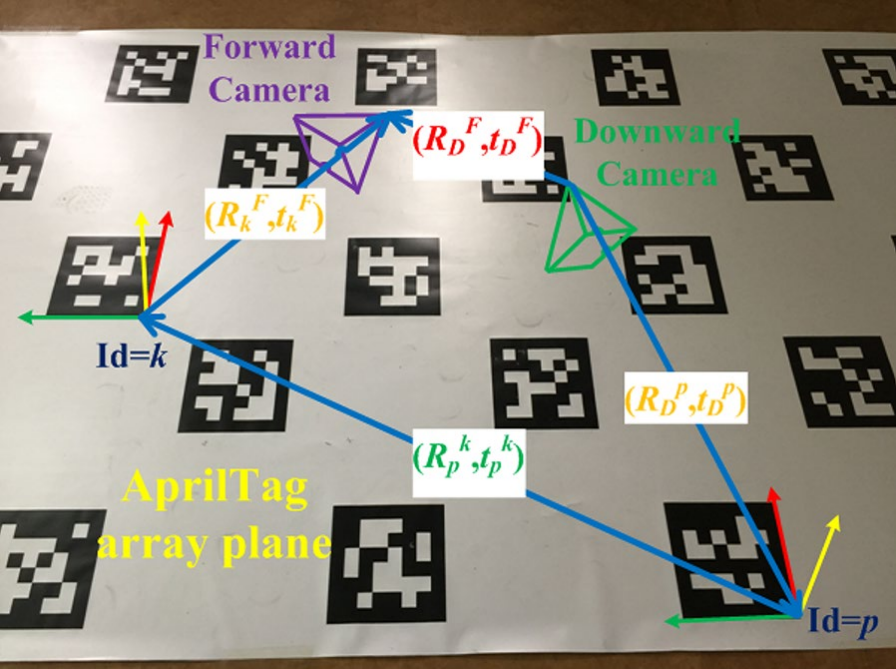
Diagram of dual-camera extrinsic calibration. The downward camera and forward camera are located in tag coordinate systems whose IDs are *p* and *k*, respectively. We can compute the extrinsic parameters (*R*_*D*_^*F*^, *t*_*D*_^*F*^) by Euclidean transformation matrixes multiplication as formula (2)

We divide all the cameras into neighboring camera pairs (see Fig. 3) and then estimate all the camera extrinsic parameters through extrinsically calibrating all the neighboring camera pairs. Hence, the main problem is how to estimate the extrinsic parameters between the camera pairs. According to the diagram of the dual-camera extrinsic calibration (Fig. 4), the closer to 180° the angle *θ* between their optical axes is, the larger the AprilTag array needs to be for the condition that at least one complete AprilTag needs to be in the field of view of each camera. More importantly, the angle *Φ* between the AprilTag’s normal vector and the vector to the camera affects the localization accuracy [3], and without loss of generality, the *Φ* only depends on the *θ*. Therefore, the *θ* should be the most important reference to the camera pairs grouping in principle.

#### Dual-camera extrinsic calibration

Figure 4 presents the diagram of the dual-camera extrinsic calibration. The two cameras, forward camera and downward camera, can be located in different tag coordinate systems, which means that the (*R*_*D*_^*P*^, *t*_*D*_^*P*^) and (*R*_*k*_^*F*^, *t*_*k*_^*F*^) are provided by AprilTag [3] directly. *R*_*D*_^*P*^ and *R*_*k*_^*F*^ stand for rotation matrixes from downward camera coordinate system to tag (ID = *p*) coordinate system; *t*_*D*_^*P*^ and *t*_*k*_^*F*^ denote the translation vectors. The purpose of this camera extrinsic calibration is to estimate (*R*_*D*_^*F*^, *t*_*D*_^*F*^). According to the Euclidean transformation [18] and formula (1), the extrinsic parameters of the dual cameras can be obtained by the Euclidean transformation matrixes’ multiplication:

2 $$T_{D}^{F} = T_{D}^{p} T_{p}^{k} T_{k}^{F}$$

where

$$T_{k}^{F} = \left( {T_{F}^{k} } \right)^{ - 1} = \left[ {\begin{array}{*{20}l} {R_{F}^{k} } \hfill & \quad {t_{F}^{k} } \hfill \\ {0_{1 \times 3} } \hfill & \quad 1 \hfill \\ \end{array} } \right]_{4 \times 4}^{ - 1}$$

and *T*_*D*_^*p*^, *T*_*p*_^*k*^, *T*_*k*_^*F*^ denote corresponding Euclidean transformations. It should be noted that the *T*_*p*_^*k*^ can be computed conveniently because all the tag coordinate system transformations are modeled in advance.

#### Single-camera localization optimization

It is possible that there are several complete tags appearing in the field of view of a single camera at the same time. The AprilTag would provide us several poses relative to these detected tags. These poses may not be same after the transformations into global coordinate system for the reason that the pose estimation error in different tag coordinate systems is different. Obviously, these measurement errors affect the performance of the extrinsic calibration.

The origin *Q*_*o*_^*G*^ = [0, 0, 0]^*T*^ of the global coordinate system can be represented in the tag (ID = *i*) coordinate system as *Q*_*o*_^*i*^. Then, we can transform it into camera coordinate system:

$$Q_{oi}^{C} = R_{i}^{C} Q_{o}^{i} + t_{i}^{C}$$

and we can transform the origin *Q*_*o*_^*G*^ into camera coordinate system directly:

$$Q_{o}^{C} = \left( {R_{C}^{G} } \right)^{ - 1} \left( {Q_{o}^{G} - t_{C}^{G} } \right) = - \left( {R_{C}^{G} } \right)^{ - 1} t_{C}^{G}$$

where *R*_*C*_^*G*^ and *t*_*C*_^*G*^ are the extrinsic parameters of a single camera. We can refine them by a minimization on a Euclidean distances function *F*(*R*_*C*_^*G*^, *t*_*C*_^*G*^):

3 $$F\left( {R_{C}^{G} ,t_{C}^{G} } \right) = \sum\limits_{{}}^{n} {\left\| {Q_{oi}^{C} - Q_{o}^{C} } \right\|}^{2} = \sum\limits_{{}}^{n} {\left\| {R_{i}^{C} Q_{o}^{i} + t_{i}^{C} + \left( {R_{C}^{G} } \right)^{ - 1} t_{C}^{G} } \right\|}^{2}$$

The function above assumes that there are *n* tags detected in image. *R*_*n*_^*C*^ and *t*_*n*_^*C*^ denote tag (ID = *n*) rotation matrix and translation vector, respectively, relative to the camera, which are given by AprilTag. We model the *F*(*R*_*C*_^*G*^, *t*_*C*_^*G*^) minimization as a nonlinear optimization problem and figure it out by using the Levenberg–Marquardt method [19, 20].

### Camera and laser extrinsic calibration

It is assumed that there is at least one camera sharing common field of view with the 2D laser scanner, which means that the laser points are in the field of view of that camera. We extrinsically calibrate the 2D laser scanner and the camera using plane-line correspondence. Unlike [14, 21], our method employs the AprilTag array to be the calibration target.

Denote the coordinates of a point with respect to the 2D laser scanner coordinate system and camera coordinate system by *Q*^*L*^ and *Q*^*D*^. The coordinates are related as follows:

$$Q^{D} = R_{L}^{D} Q^{L} + t_{L}^{D}$$

where *R*_*L*_^*D*^ and *t*_*L*_^*D*^ represent rotation matrix and translation vector from laser scanner coordinate system to camera coordinate system. The estimation of (*R*_*L*_^*D*^, *t*_*L*_^*D*^) is the purpose of this calibration.

**Fig. 5:**
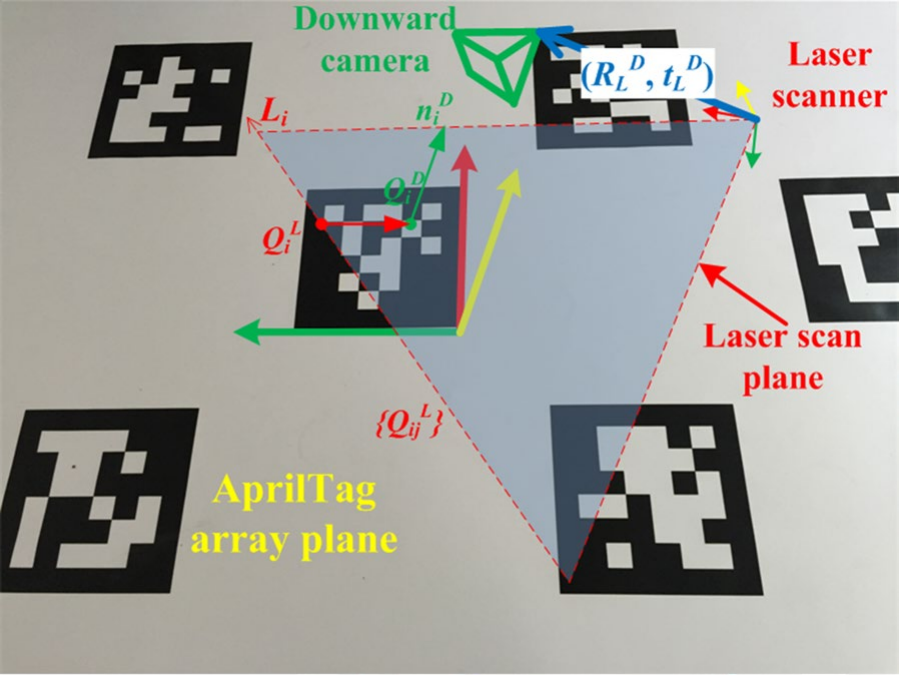
Diagram of camera and laser extrinsic calibration. The extrinsic parameters can be computed by plane-line correspondence as formula (4)

 As Fig. 5 shows, the AprilTag array is placed in the common field of view of the camera and laser scanner. We can compute the normal vector *n*_*i*_^*D*^ of the AprilTag array in the camera coordinate system through single-camera extrinsic calibration in previous section. There is an intersection line *L*_*i*_ (3 × 1 vector in laser scanner coordinate system) of the AprilTag array and laser scan plane, on which laser scanner provides many discrete points {*Q*_*ij*_^*L*^}. According to the plane-line correspondence, two geometry constraints on (*R*_*L*_^*D*^, *t*_*L*_^*D*^) are given:

$$\left( {n_{i}^{D} } \right)^{T} R_{L}^{D} L_{i} = 0$$

4 $$\left( {n_{i}^{D} } \right)^{T} \left( {R_{L}^{D} Q_{i}^{L} + t_{L}^{D} - Q_{i}^{D} } \right) = 0$$

*Q*_*i*_^*D*^ is the 3D coordinate of a point in AprilTag array plane with respect to the downward camera coordinate system. There are 6 degrees of freedom in *R*_*L*_^*D*^ and *t*_*L*_^*D*^ together. Hence, to solve above equations, three correspondences are required at least. The whole process includes linear solution and nonlinear optimization. The nonlinear optimization is a nonlinear minimization process on the Euclidean distances from laser points to the AprilTag array plane. The error function is defined as:

$$\sum\limits_{i} {\sum\limits_{j} {\left( {\frac{{n_{i}^{D} }}{{\left\| {n_{i}^{D} } \right\|}}\left( {R_{L}^{D} Q_{ij}^{L} + t_{L}^{D} } \right) - \left\| {n_{i}^{D} } \right\|} \right)^{2} } }$$

where ||*n*_*i*_^*D*^|| equals the distance from camera to the calibration plane. This error function is minimized by the Levenberg–Marquardt method [19, 20]. The details of the linear solution, nonlinear optimization and global optimization are described in [14].

### Global optimization

As mentioned in previous section, we divide the multiple-sensor extrinsic calibration into two independent processes, but they can be finished in a common procedure. Simultaneously, a closed-loop optimization can be used to refine the extrinsic parameters obtained by the two processes. Assuming that the downward camera and the laser scanner have a common field of view and the *T*_*D*_^*F*^ and *T*_*L*_^*D*^ have been calibrated, *T*_*L*_^*F*^ can be computed as:

$$T_{L}^{F} = T_{L}^{D} T_{D}^{F} \left\lfloor {\begin{array}{*{20}l} {R_{L}^{D} R_{D}^{F} } \hfill & \quad {R_{L}^{D} t_{D}^{F} + t_{L}^{D} } \hfill \\ {0_{3 \times 3} } \hfill & \quad 1 \hfill \\ \end{array} } \right\rfloor_{4 \times 4}$$

Although the forward camera and the laser scanner may have no common field of view, we can still refine *T*_*L*_^*F*^ by the nonlinear minimization process described in previous section.

**Fig. 6:**
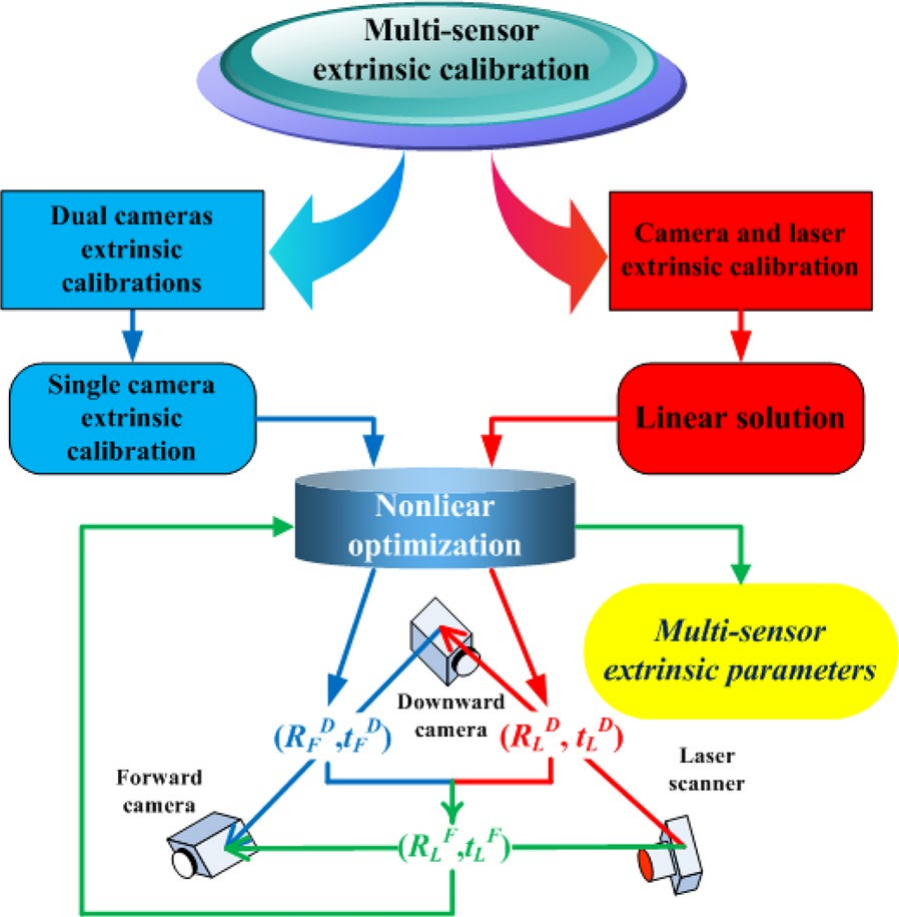
Schematic of the multi-sensor system extrinsic calibration algorithm. It is assumed that the two cameras have no overlapping field of view, and the downward camera and laser scanner have common field of view

Figure 6 shows the whole calibration algorithm schematic, and the details are described as the following workflow:

**Table:** 

**Algorithm**: Multi-sensor system extrinsic calibration
**Inputs**:
1. Simultaneous images and laser scan points’ coordinates.
2. Intrinsic parameters of each camera.
3. The transformations between two arbitrary tags in the calibration target.
**Procedure**:
1. Dual-camera extrinsic calibration
(a) Single-camera extrinsic calibration using AprilTag.
(b) Optimization of single-camera extrinsic parameters by a nonlinear minimization process.
(c) Computing the extrinsic parameters (*R*_*D*_^*F*^, *t*_*D*_^*F*^) by the matrix transformations.
2. Camera and laser extrinsic calibration
(a) Using plane-line correspondence for the geometry constraints.
(b) Solving these equations for the linear solution.
(c) Optimizing the linear solution to refine the camera and laser extrinsic parameters.
3. Global optimization to refine the multi-sensor system extrinsic parameters.
(a) Computing the extrinsic parameters between other cameras and the laser.
(b) Global optimization of all the sensors extrinsic parameters.
**Output**: Extrinsic parameters of multi-sensor system

## Experiments

### Single-camera pose estimation

**Fig. 7:**
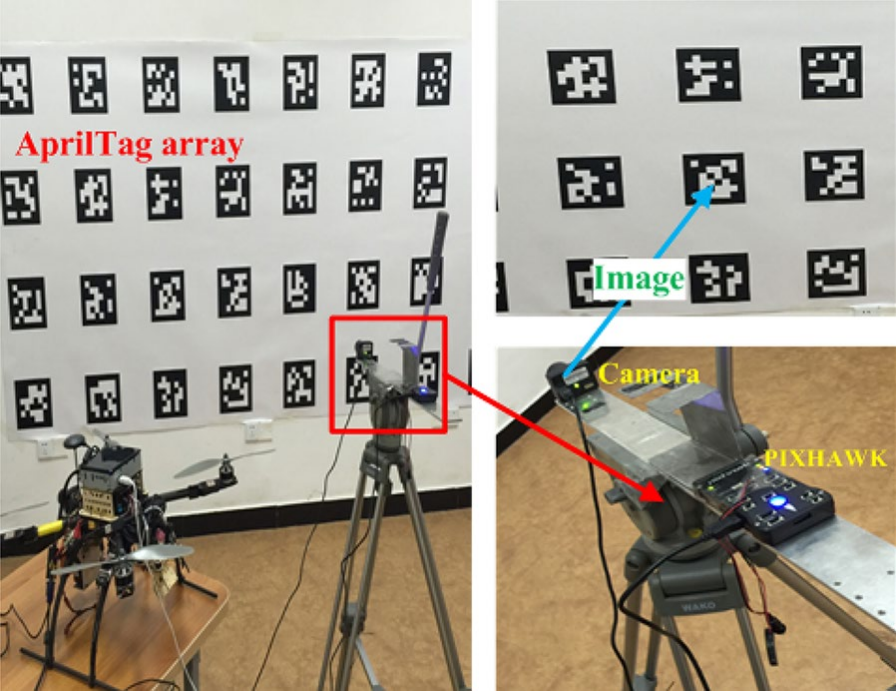
Single-camera pose estimation experiment. The PIXHAWK provides the real camera attitude. The *upper right* image is captured by the camera

As description of the camera–laser extrinsic calibration, the normal vector *n*_*i*_^*D*^ is obtained by single-camera pose estimation. Therefore, the dual-camera extrinsic calibration and camera–laser extrinsic calibration significantly depend on the single-camera pose estimation. To estimate the camera pose with higher accuracy, a nonlinear optimization process is employed when multiple tags are in the field of view of the single camera (see Fig. 7). The calibration pattern plane is pasted on the wall, which is defined by 10 × 4 tags with 0.2 m × 0.2 m size of each tag and 0.2 m space between two tags. We mounted a camera (Point Grey FireFly MV) and a PIXHAWK on a tripod. The camera attitude can be measured by the IMU module in PIXHAWK. These layouts make us conveniently locate the camera in calibration target coordinate system using some measuring devices, and the measurements are regarded as the ground truth.

**Fig. 8:**
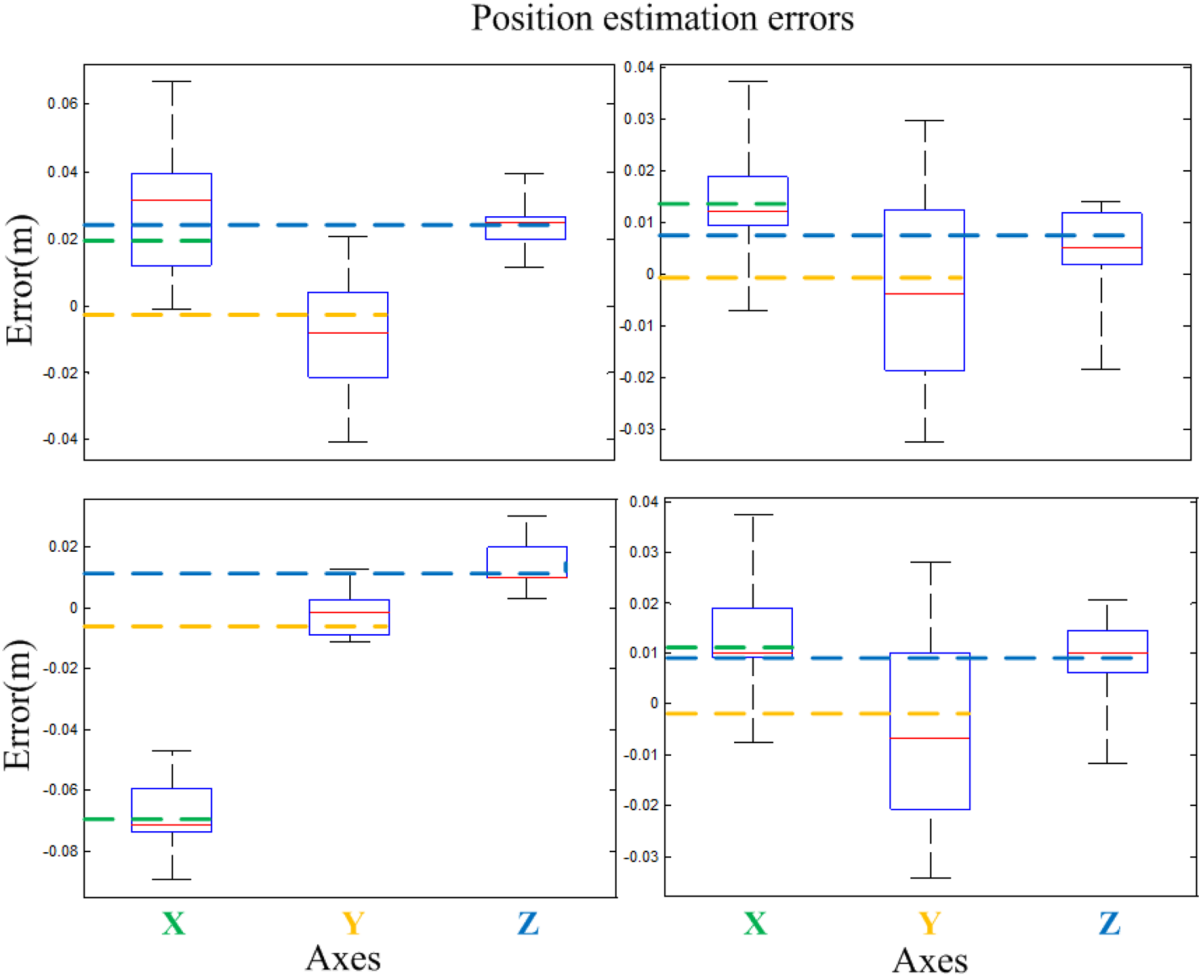
Four sets of experiment results of the position estimation errors in *X*, *Y* and *Z* axes, respectively, using multiple tags detected in the common field of view. The optimization result errors are denoted as the *dotted line* with corresponding *colors* of axes

Figure 7 shows the experiment scene. In one of the experiments, there are about 8 complete tags in the field of view of the camera. We record all the poses provided by each tag and use the nonlinear optimization algorithm to compute the camera final pose in global coordinate system. Four sets of data are collected, and we compute the position estimation errors in *X*, *Y* and *Z* axes, respectively, by each tag. Figure 8 presents the errors distribution and the final pose with nonlinear optimization process. The root-mean-square errors (RMSEs) are computed to be compared with the optimization result errors, and the results are presented in Table 1. Obviously, this optimization shows a better position estimation accuracy when multiple tags are in the field of view.

**Table 1:** Position estimation of RMSE with multiple estimations by multiple tags

Index	Axes	Multiple tags (RMSE)	Optimization results
1	*X* (m)	0.034	0.020
	*Y* (m)	0.020	0.003
	*Z* (m)	0.025	0.021
2	*X* (m)	0.020	0.013
	*Y* (m)	0.018	0.001
	*Z* (m)	0.009	0.008
3	*X* (m)	0.069	0.069
	*Y* (m)	0.008	0.008
	*Z* (m)	0.017	0.010
4	*X* (m)	0.018	0.011
	*Y* (m)	0.019	0.002
	*Z* (m)	0.012	0.009

We use the IMU to measure the yaw angles as the ground truth because the camera, mounted on the tripod, is rotated at yaw direction, and the experiment results show that the pitch and roll angles are closed to 0. From Table 2, we can see that the yaw angle estimation errors are decreased by the optimization process. In summary, these experiments above show higher pose estimation accuracy with the nonlinear optimization when multiple tags are in the field of view.

**Table 2:** Attitude estimation of RMSE with multiple tags and the optimization result errors

Index	Axes	Multiple tags (RMSE)	Optimization results
1	Yaw (°)	2.8339	1.9307
	Pitch (°)	0.4180	0.4491
	Roll (°)	0.9443	0.7802
2	Yaw (°)	2.7023	1.8842
	Pitch (°)	0.3993	0.5832
	Roll (°)	0.4032	0.3400

The attitude is represented as Euler angles

### Multi-sensor jointly extrinsic calibration

**Fig. 9:**
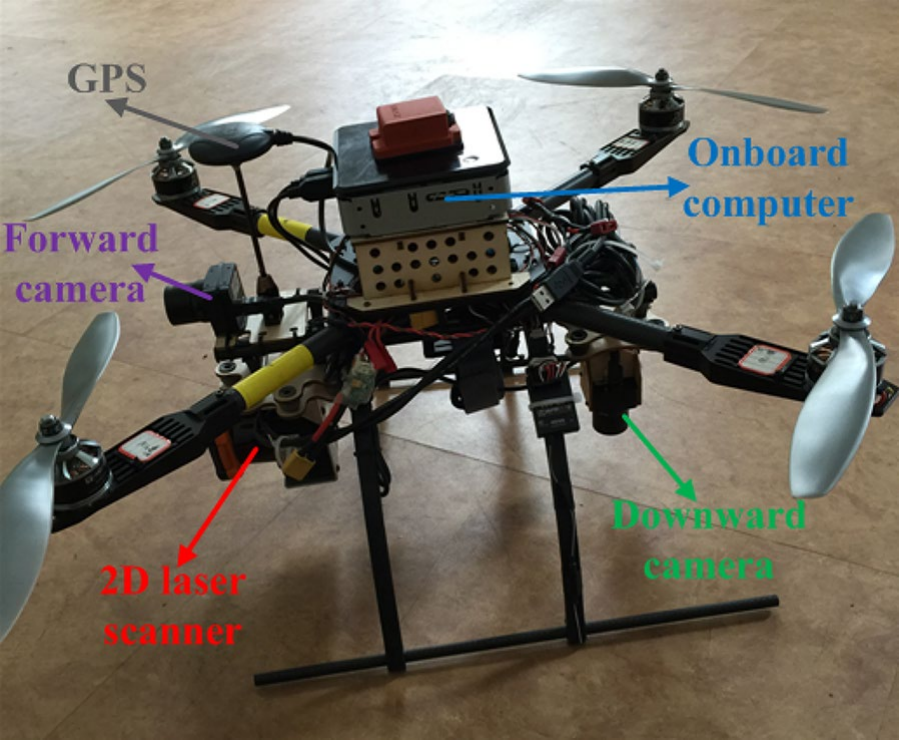
Onboard multi-sensor system for validation of this calibration algorithm with corresponding colors of axes

The proposed algorithm has been implemented in ROS (Robot Operating System) and tested on real data with a of a rotary-wing UAV platform (see Fig. 9). An onboard processing computer (Intel NUC D54250WYKH), a flight controller (PIXHAWK), a cameras–laser sensor module (two Point Grey FireFly MV cameras, Hokuyo UTM-30LX laser scanner) and a GPS are integrated as an onboard multi-sensor system.

**Fig. 10:**
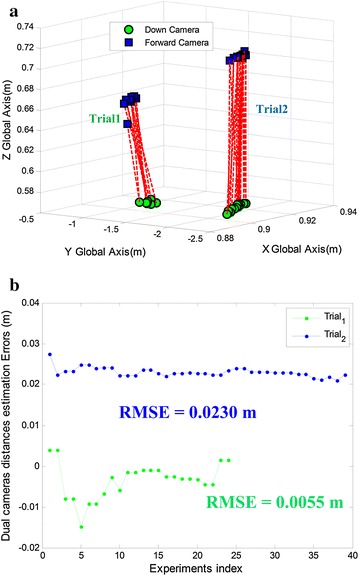
Two sets of trials of the dual-camera extrinsic calibration. The results are refined by global optimization. **a** The estimated dual-camera positions. Each dotted line connects forward camera with downward camera at the same time. **b** The dual-camera distances estimation errors. The *green* and *blue* points are corresponding to the two sets of trials, respectively, in **a**

This experiment is to extrinsically calibrate the onboard multi-sensor system and globally refine all the extrinsic parameters. With the basis of the accurate pose estimation presented in previous section, the dual-camera extrinsic calibration should be as accurate as the single-camera extrinsic calibration. Here, we run two sets of independent trials. Figure 10a shows the dual-camera calibration results after global refinement, and the two end points of each red dotted line stand for forward and downward camera positions, respectively, at the same time. It should be noted that there is a result seems to be with larger error in Trial1. Actually, it is the inappropriate perspective that leads to this misunderstanding. This result is corresponding to the first blue point in Fig. 10b, and obviously, the distance error is not so large. The distances between the dual cameras, which is the length of these dotted lines in Fig. 10a, are computed conveniently to be compared with the ground truth (see Fig. 10b). The RMSE (0.0230 and 0.0055 m) is acceptable.

**Fig. 11:**
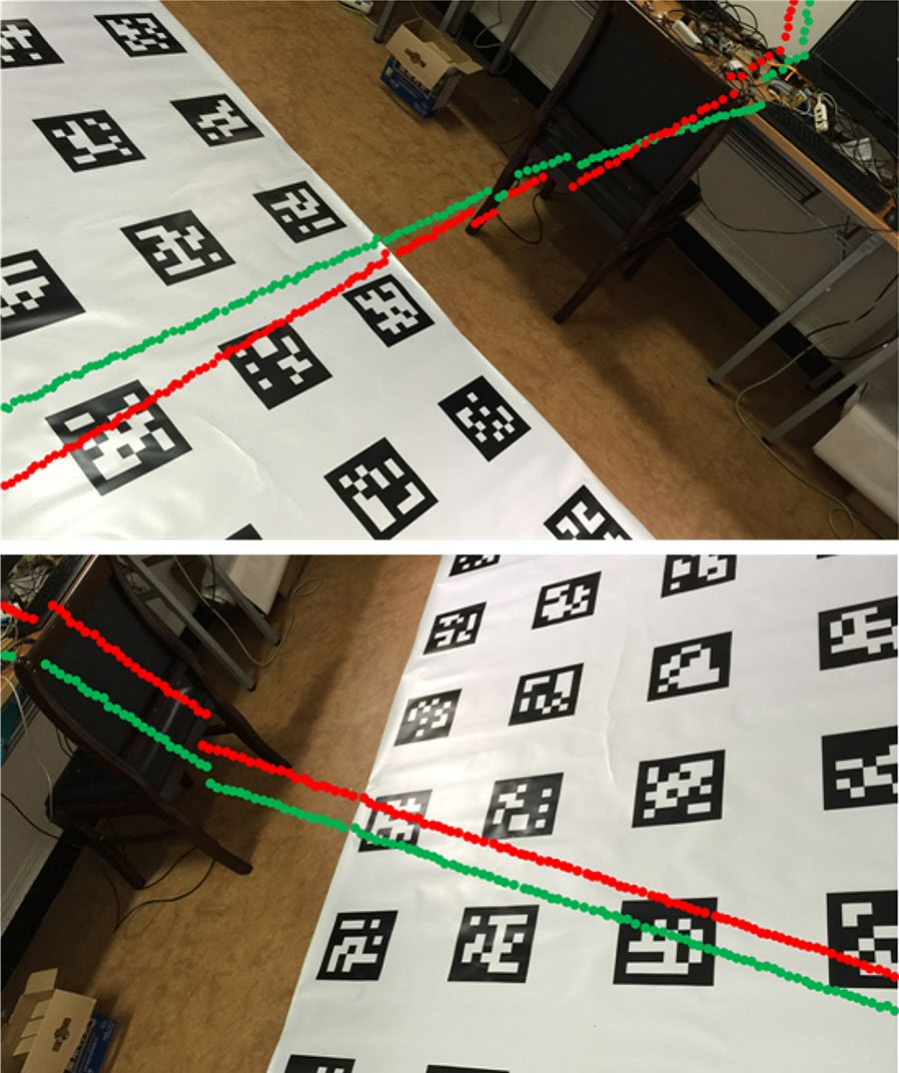
Projection of the laser points onto the images. The two images are captured in the two trials above. The *red points* and *green points* are mapped using the extrinsic parameters by our method and method in [14], respectively

Meanwhile, the downward camera and the laser scanner are extrinsically calibrated in the two sets of trials. To demonstrate a better performance of our method over the method in [14], the laser points are projected onto the images of the downward camera using the extrinsic parameters provided by both methods. Figure 11 shows the mapping results. We do not have the ground truth of the camera–laser extrinsic parameters, but the mapping results by our method (red dotted line) are more reasonable. Hence, we can conclude that our method calibrates the extrinsic parameters more accurately.

## Conclusion and future work

In this paper, we proposed a new algorithm for extrinsically calibrating multi-sensor system including multiple cameras and a 2D laser scanner using the AprilTag array as the calibration target. This algorithm uses the AprilTag to estimate cameras’ poses and employ a nonlinear optimization method to refine these poses when multiple tags are in the field of view. Then, the camera–camera and laser–camera extrinsic parameters are estimated on the basis of the single-camera pose. Finally, a global optimization is used to refine all the extrinsic parameters. This algorithm is adapted to multiple-camera extrinsic calibration with non-overlapping field of view, and it has the advantages of being simple to use and yields great performance, which have been validated by real data.

In future work, more experiments and analyses about the influences of the tags number in the field of view or the camera module, etc. on the calibration accuracy should be carried out. In addition, it should be possible to extend the multi-sensor system into other sensor configurations, and an accurate and stable dynamic calibration can be taken into account.
